# Quantitative Analysis of Nutrient Elements in Soil Using Single and Double-Pulse Laser-Induced Breakdown Spectroscopy

**DOI:** 10.3390/s18051526

**Published:** 2018-05-11

**Authors:** Yong He, Xiaodan Liu, Yangyang Lv, Fei Liu, Jiyu Peng, Tingting Shen, Yun Zhao, Yu Tang, Shaoming Luo

**Affiliations:** 1 College of Biosystems Engineering and Food Science, Zhejiang University, 866 Yuhangtang Road, Hangzhou 310058, China; yhe@zju.edu.cn (Y.H.); m15307266704@163.com (X.L.); 18143465995@163.com (Y.L.); jypeng@zju.edu.cn (J.P.); shentingtingstt@163.com (T.S.); zhaoyun@zust.edu.cn (Y.Z.); 2 Key Laboratory of Spectroscopy Sensing, Ministry of Agriculture, Hangzhou 310058, China; 3 School of information and electronic engineering, Zhejiang University of Science and Technology, 318 Liuhe Road, Hangzhou 310023, China; 4 College of Automation, Zhongkai University of Agriculture and Engineering, Guangzhou 510225, China; tangyu_mycauc@163.com (Y.T.); smluo@gdut.edu.cn (S.L.)

**Keywords:** soil, nutrient elements, laser-induced breakdown spectroscopy, single-pulse, double-pulse, chemometrics

## Abstract

Rapid detection of soil nutrient elements is beneficial to the evaluation of crop yield, and it’s of great significance in agricultural production. The aim of this work was to compare the detection ability of single-pulse (SP) and collinear double-pulse (DP) laser-induced breakdown spectroscopy (LIBS) for soil nutrient elements and obtain an accurate and reliable method for rapid detection of soil nutrient elements. 63 soil samples were collected for SP and collinear DP signal acquisition, respectively. Macro-nutrients (K, Ca, Mg) and micro-nutrients (Fe, Mn, Na) were analyzed. Three main aspects of all elements were investigated, including spectral intensity, signal stability, and detection sensitivity. Signal-to-noise ratio (SNR) and relative standard deviation (RSD) of elemental spectra were applied to evaluate the stability of SP and collinear DP signals. In terms of detection sensitivity, the performance of chemometrics models (univariate and multivariate analysis models) and the limit of detection (LOD) of elements were analyzed, and the results indicated that the DP-LIBS technique coupled with PLSR could be an accurate and reliable method in the quantitative determination of soil nutrient elements.

## 1. Introduction

Soil is the foundation of crop growth, and plays an important role in the whole ecosystem [1]. In particular, soil nutrient elements, including macro-nutrients and micro-nutrients, are the basic resources for preserving the surface ecosystem. Meanwhile, the content of nutrient elements can reflect the fertility of the soil, and are considered an important index of crop yield evaluation [2]. It is particularly important to obtain the content of soil nutrient elements quickly and accurately for agricultural production. Currently, the main methods for the detection of nutrient elements in soil include laboratory chemical analysis, such as atomic absorption spectrometry (AAS) [3]; inductively-coupled plasma optical emission spectrometry (ICP-OES) [4]; and inductively-coupled plasma mass spectrometry (ICP-MS) [5]. However, these traditional chemical detection methods are time-consuming, complex, and cannot meet the demands of real-time detection.

Laser-induced breakdown spectroscopy (LIBS) is a recently developed spectral detection technique [6]. Because of the advantages of fast analytical speed, simple sample pretreatment, multi-element simultaneous detection capability, as well as the potential of in situ or stand-off analysis [7], it has been widely used for the elemental analysis of various kinds of samples, including plants [8], water [9], and especially soils. Martin et al. applied LIBS to determine the carbon and nitrogen content of various soils, and the good results proved that LIBS technique had the potential to be packaged into a field-deployable instrument for real-time and in situ analysis of soil carbon and nitrogen [10]. Harris et al. presented their investigation on LIBS detection of nitrogen in sand at atmospheric and reduced pressures. They found that a pressure below 0.04 torr air could be feasible for nitrogen monitoring in sand or soil [11]. Dong et al. also reported on LIBS detection of nitrogen in farmland soil. A good correlative relationship between nitrogen content and LIBS signal intensity was obtained by using the adoption of buffer gases, which provided guidance for the development of the real-time farmland nitrogen measurement system [12]. Yang et al. used LIBS coupled with chemometrics methods to quantitatively analyze Cu and Zn concentrations in soil samples, and a good performance was obtained using the PLS calibration model, with R^2^ reaching 0.94 in prediction set for both Cu and Zn. The results indicated that LIBS combined with PLS could be a good method for the soil analysis [13]. Popov et al. studied the possibility of quantitative detection of Zn in soils using LIBS, and the limit of detection (LOD) of Zn reached 18 ppm, which was far below Occupational Exposure Limits (OEL) in soil (150 ppm) and the mean abundance of Zn in the earth’s crust (83 ppm) [14]. Awasthi et al. employed LIBS combined with PLSR to analyze environmental reference materials (RMs), and the predicted concentrations of elements (Al, Ca, Mg, Fe, K, Mn and Si) by LIBS are comparable to the certified concentrations. The results demonstrated the ability of LIBS for rapid analysis of RMs [15]. It is also worth noting that the element analysis mentioned above depends on the information of the atomic or ionic spectra in the plasma, which is generated by the laser pulse ablation of samples [16]. At present, single-pulse (SP) and collinear double-pulse (DP) are the most commonly used methods for ablation of samples [17,18,19]. SP-LIBS has one laser beam to ablate samples, while two laser beams with delay time are used in collinear DP-LIBS; one for sample ablation, and the other for plasma reheating [20]. In recent years, several studies concerning on the detection of elements contents using SP-LIBS and DP-LIBS have been published. Liu et al. used SP-LIBS combined with chemometrics methods to detect the copper content in rice and obtained a good result using a partial least squares regression (PLSR) model, with R^2^ more than 0.97 in both calibration and prediction set, and the LOD reached as low as 1.95 ppm [21]. Afgan et al. focused on the determination of the elements Si, Cr, Mn and Ni in various steel samples using a handheld SP-LIBS instrument. The average relative standard deviation (RSD) for these elements was less than 5%, indicating that LIBS is a practical method for the fast detection of elements [22]. Pedarnig et al. employed SP-LIBS and DP-LIBS to analyze the halogen chlorine (Cl) in industrial iron oxide. It was proved that DP-LIBS had very high excited state energies, which contributed to sensitive detection of Cl and other elements [23]. Kwak et al. reported on the quantitative analysis of arsenic (As) in mine tailing soils using DP-LIBS. The results showed that the use of DP-LIBS enhanced the intensity and signal-to-noise ratio (SNR) of emission lines, while decreasing the value of RSD. Furthermore, it provided a good quantification performance, with a correlation coefficient of 0.94 [24]. To the best of our knowledge, the quantitative detection of soil nutrient elements simultaneously based on SP-LIBS and collinear DP-LIBS haven’t been investigated.

The purpose of this study was to explore the detection ability of SP-LIBS and collinear DP-LIBS for soil nutrient elements, and to find a practical method for fast and accurate quantitative detection. In this work, spectral intensity and signal stability of all elements for SP and DP signals were directly compared. Chemometrics methods including the univariate method, PLSR and least-squares support vector machine (LS-SVM) were employed to establish the calibration model for quantitative analysis of elements. The detection sensitivity for SP-LIBS and collinear DP-LIBS was further compared based on the performance of chemometrics models.

## 2. Materials and Methods

### 2.1. Soil Samples

The certified reference material (CRM) of soil tested in this research was provided by National Institute of Metrology, China, and 6 classes were involved. These were: sandy soil (GBW07446) from Inner Mongolia Autonomous Region, sediment (GBW07452) from the Xiangshan east beach in zhejiang province, sediment (GBW07453) from the Yangjiang south beach in Guangdong province, loess (GBW07454) from Shaanxi province, sediment (GBW07455) from the Huaihe River in Anhui province, and sediment (GBW07456) from the Zhangjiagang chengjiang River in Jiangsu province. All soil powders were sieved through a 100-mesh screen, and then dried at 60 °C for 4 h in an oven. In order to get more concentration gradient to establish stable quantitative analysis models, we took 6 certified soil samples and 15 mixed soil samples as our objects. Specifically, 0.25 g powders from each of 6 certified soil samples was weighed and mixed at the ratio of 1:1. Additionally, 0.5 g powders from each of 6 certified soil samples was weighed. All soil powders were pressed into tablets with 12 mm in diameter and 2 mm in thickness, using 10-MPa force for 60 s (FY-24, SCJS, Tianjin, China). The sample of each concentration was repeated three times. Thus, 63 soil tablets were prepared for SP experiment and collinear DP experiment, respectively. In addition, according to certified elemental compositions of the oxide material (K_2_O, CaO, MgO, FeO, Mn, Na_2_O) in soil CRM, measured by the Institute of Geophysical and Geochemical Exploration of the Chinese Academy of Geological Science, we calculated the concentrations of major nutrient elements (K, Ca, Mg, Fe, Mn, Na), listed in Table 1.

### 2.2. Spectral Acquisition

**Figure 1:**
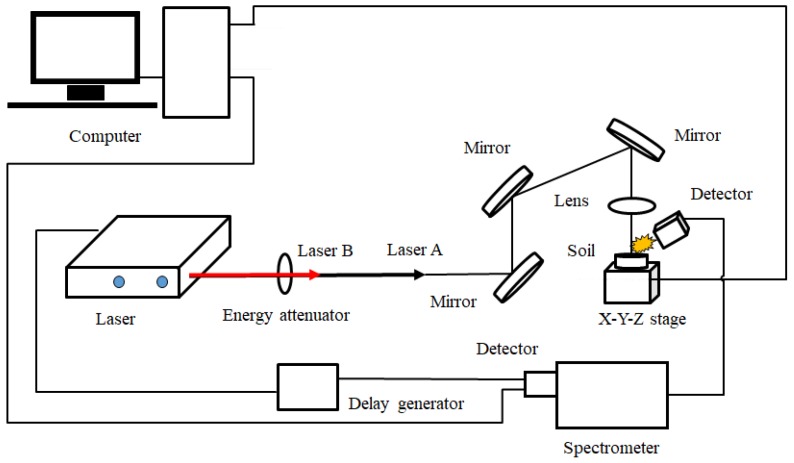
Schematic diagram of LIBS system for soil samples.

The spectral acquisition system used in this experiment was presented in Figure 1. Q-switched Nd:YAG pulsed laser (Vlite-200, Beamtech Optronics, Beijing, China) was used for generating laser pulses, operated at 532 nm. An Echelle spectrograph (ME5000, Andor Technology, Belfast, UK) was used for dispersion of spectral emission lines. An intensified charge-coupled device (ICCD) camera (iStar DH340T, Andor Technology, Belfast, UK) was applied for detection of the optical signal. A digital delay generator (DG645, Stanford Research Systems, San Jose, CA, USA) was used to control the delay time between the laser and the ICCD camera, and the delay time between two lasers in DP system. Lens, mirrors, and optics were used for laser transmission and beam guiding. For the SP experiment, only laser A was needed, while laser A and B were transmitted in the same line with a pulse interval in the collinear DP experiment. Before the experiment, we optimized the experimental parameters based on the response surface methodology (RSM). As a result, under the conditions of SP, the parameters of laser energy, delay time and gate width were optimized at 100 mJ, 6 µs and 10 µs, respectively. Compared with the SP-LIBS experiment, the collinear DP-LIBS experiment has more parameters, including laser energy for the first beam, laser energy for the second beam, delay time, gate width and pulse interval, which were optimized at 25 mJ, 75 mJ, 6 µs, 10 µs and 1.2 µs, respectively. After setting parameters, we placed the sample on the X-Y-Z stage to avoid continuous ablation of the same spot. In addition, in order to get a stable signal, the laser beam was focused 2 mm below the sample surface. For each pellet, a total of 16 spots were struck, with continuous shots 5 times for each spot. 

### 2.3. Data Analysis

#### 2.3.1. Data Preprocessing

Considering the matrix effect of the sample, and the systematic and random errors during the experiment, we used spectral preprocessing methods to eliminate the influence and improve the accuracy of data analysis. Data average means to replace the spectral value of the sample with the accumulated average of multiple spectra, which could eliminate the obvious matrix effect. The wavelet transform (WT) approach, as an efficient denoising method, was used to reduce the noise generated by the instrument and the sampling process [25]. Daubechies 4 with decomposition scale 3 was optimized when the maximal SNR was obtained; detailed information can be found in the literature [26]. In addition, area normalization was used to decrease the matrix effect and spectral error caused by the change of experimental parameters.

#### 2.3.2. Chemometrics Methods

Univariate analysis, also called the calibration curve method, is a traditional quantitative analysis method in LIBS analysis. When the concentration of the analytical element is low and there is no self-absorption, the spectral intensity is proportional to the element content in the sample [27]. Thus, the calibration curve of the element can be fitted according to the intensity of the detected LIBS signal and the concentration of the corresponding element, and the concentration of an unknown sample can be calculated by the calibration curve. However, the method has proved to be limited with matrices, so it is more suitable for the analysis of CRM [16]. We employed this method for analysis since the experimental samples were CRM soils. In addition, the coefficient of determination for calibration (R^2^_C_) and prediction (R^2^_P_), the root mean squared error for calibration (RMSEC) and prediction (RMSEP), and limit of detection (LOD) were adopted to evaluate the performance of the univariate model. 

Partial least-squares regression (PLSR), as a classical linear modeling method, is widely used for quantitative analysis of spectra [28]. In particular, this method is designed to reflect spectral information with the first few latent variables [29,30]. Thus, the selected latent variables are critical, and directly determine the predictive performance of the model. In this study, we tried to establish the PLSR model of the concentration of nutrients and signal intensity of SP and DP for soil samples. In order to obtain a robust and reliable model, and to avoid the overfitting of the model, full cross-validation was applied, and the number of latent variables was determined when the minimal mean squared error was obtained. Additionally, evaluation indexes of the univariate model were also applicable to the PLSR model.

Least-squares support vector machine (LS-SVM) is a supervised machine learning method based on the standard SVM, which is especially suitable for classification and regression of a small number of samples [31,32,33]. Herein, the radial basis function (RBF) kernel function was utilized to establish the LS-SVM model, due to the good nonlinear solving ability [34,35]. A grid-search procedure in the range of 10^3^–10^10^ was carried out to optimize parameters, and the best penalty parameters (c) and kernel function parameters (g) were determined when the value of RMSECV reached its minimum. Full cross-validation was also conducted to avoid the overfitting of the model. A detailed introduction to the LS-SVM model can be found in these references: [36,37]. In addition, evaluation indicators of the performance of the model were the same as those of the univariate model and the PLSR model. 

#### 2.3.3. Performance Evaluation

Signal-to-noise ratio (SNR) and relative standard deviation (RSD) are important indices for measuring signal sensitivity and stability in spectral analysis [38]. In detail, SNR is the ratio of net analytical signal to noise interference, and RSD is the ratio of standard deviation of signal to average intensity of signal. Moreover, the greater the SNR is, the better the instrument’s detection ability will be. The smaller the RSD is, the more stable the collected signal will be. Thus, we applied these two indices to compare the stability of SP and collinear DP signals.

In terms of element detection, the performance of the calibration model plays a crucial role in the evaluation of detection sensitivity. Thus, evaluation indicators of chemometrics models can be further applied to compare the detection sensitivity of the SP-LIBS and collinear DP-LIBS. Specifically, the greater the values of R^2^_C_ and R^2^_P_, the better the detection sensitivity. The smaller the values of RMSEC and RMSEP, the higher the accuracy of the detection. LOD is also a key indicator of evaluation [39]. Generally, a lower LOD represents a better detection system. The indicators of the univariate model could be calculated with the following equation:

(1) $$\mathrm{LOD}=\frac{3\sigma_{background}}{b}$$

where *σ_background_* means the standard deviation of the background intensities, *b* means the slope of calibration curve. In contrast to the univariate model, the calculation method of LOD in the PLSR model is more complicated. Detailed derivations can be found in the reference [40]. According to the derivation process, we found that *Beta Coefficients (a)* in the results of PLSR model carried out in Unscrambler X 10.1 could be used to calculate LOD. The value of LOD could be simplified as the inverse of *Beta Coefficients (a).* However, the calculation method of LOD in the LS-SVM model needs to be further studied.

### 2.4. Software Tools

Parameter optimization was performed in the software Design Expert (ver. 8.05, CAMO AS, Oslo, Norway). LIBS data acquisition was conducted in Andor SOLIS for Imaging (v4.26, Andor Technology, Belfast, UK). Data analysis was carried out by Unscrambler X 10.1 (CAMO, Process, AS, OSLO, Norway) and MATLAB R2009a (v7.8, The MathWorks, Inc., Natick, MA, USA). Additionally, Origin Pro 8.0 SR0 (Origin Lab Corporation, Northampton, MA, USA) was used for graphs designing.

## 3. Results and Discussion

### 3.1. Spectral Analysis

Referring to the National Institute of Standards and Technology (NIST) database and relevant references, several common spectral lines of each element were chosen according to the characteristic wavelengths of our experimental system, which are listed in Table 2.

**Figure 2:**
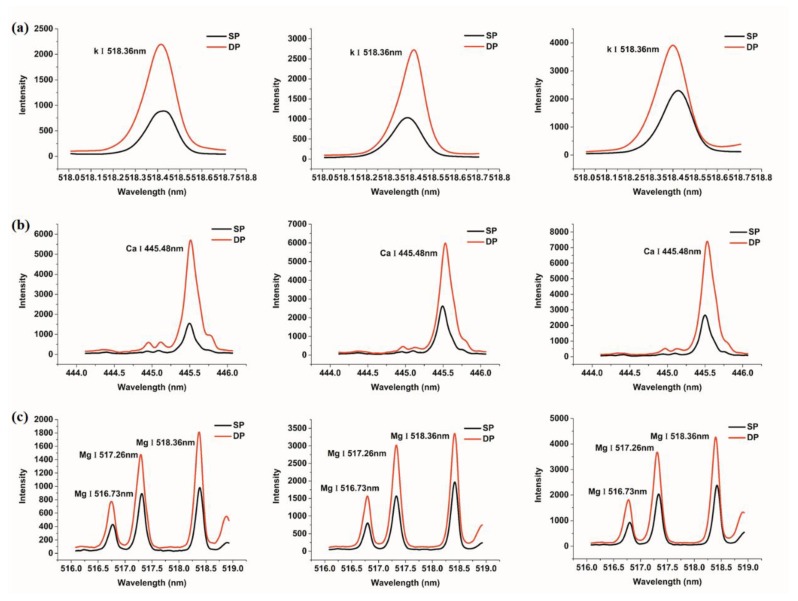

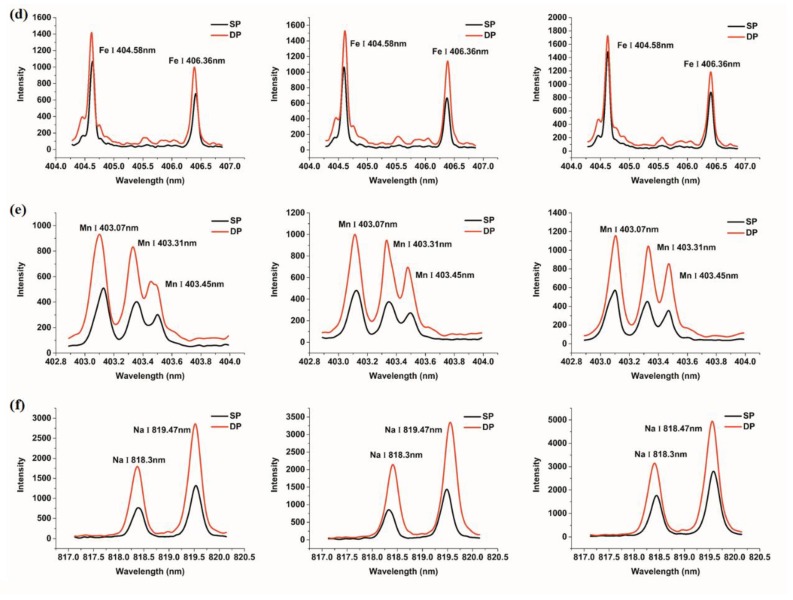
Single-pulse (SP) and double-pulse (DP) lines of soil samples with different concentrations of nutrient elements; the element concentrations from left to right are low, medium and high, respectively. (**a**) K; (**b**) Ca; (**c**) Mg; (**d**) Fe; (**e**) Mn; (**f**) Na.

A comparison of the spectral intensities of each element under the conditions of SP and collinear DP is presented in Figure 2. Considering that the spectral intensity is relevant to the concentration of elements, we compared the spectral intensities at different element concentrations. In view of the multiple lines of an element, we also took the difference of spectral lines into account, and found that they had a similar trend with respect to the intensity of the SP and DP spectral lines. Thus, we took only one line of the element as an example. As seen in Figure 2, macro-nutrients (K I 518.36 nm, Ca I 445.48 nm, Mg I 517.26 nm) and micro-nutrients (Fe I 404.58 nm, Mn I 403.07 nm, Na I 819.47 nm) were analyzed. For all elements, the intensity of collinear DP signals was stronger than that of SP signals. For SP analysis, the signal intensities of K, Fe, Mn and Na for samples with low element concentrations were similar to those for samples with medium element concentrations; meanwhile, for DP analysis, these signal intensities for samples with medium element concentrations were significantly stronger than those for samples with low element concentrations. For Ca, the emission line for medium and high concentrations could not be distinguished with SP, but could be discriminated with DP. These results indicate that collinear DP can enhance spectral signal of elements and make quantitative analysis better. 

### 3.2. Stability Analysis

**Figure 3:**
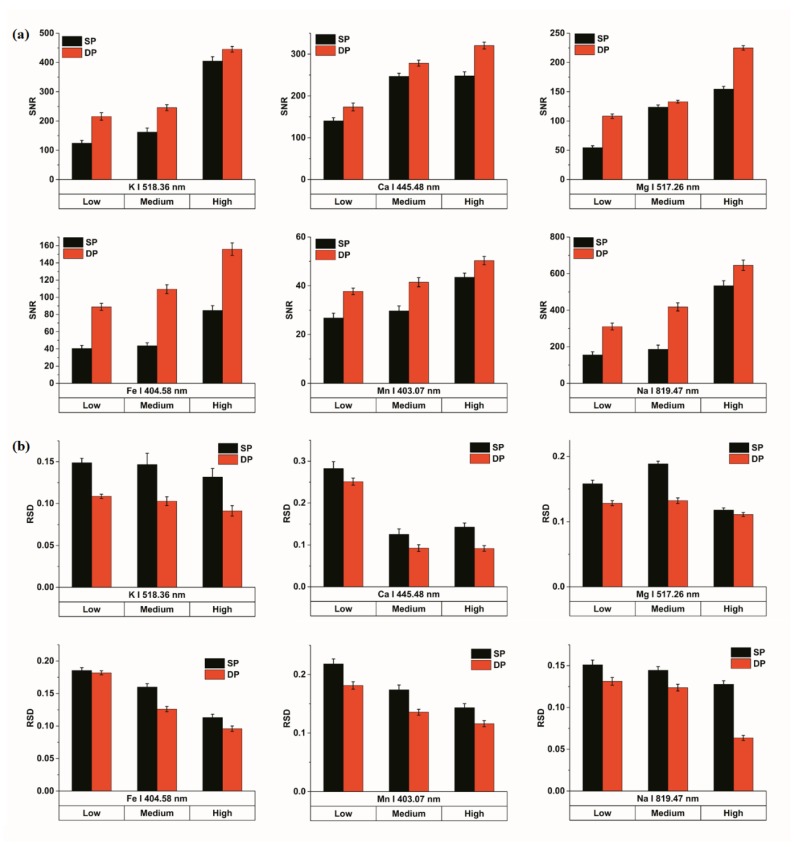
Stability analysis of elements’ SP and collinear DP signals in different sample concentrations. (**a**) Comparison of SNR; (**b**) Comparison of RSD.

Repeatability, as one of the most important indicators of experiments, is usually affected by signal stability [48]. In this study, the signal stability of SP and collinear DP signals of each element was investigated after the comparison of the spectral intensity. Specifically, SNR and RSD were chosen for evaluating signal stability. The values of SNR and RSD for the element lines of all samples were calculated. In addition, we conducted this calculation on the raw spectra of the elements; no preprocessing methods were used. It was found that the variation trends were similar between multiple lines of an element. Therefore, we only focused on one line, which was exactly the same as that used in the section of Spectral Analysis. In addition, the element concentration difference was also analyzed. The result is given in Figure 3. 

It was obvious that all elements had a common trend. The values of SNR of collinear DP signals were higher than those of SP signals, and the values of RSD were the opposite way around. Additionally, the value of SNR of both SP and collinear DP signals generally increased with the increase of concentrations. In detail, for SNR, the effect of collinear DP-LIBS on the elements K and Mg was obvious when the concentration was low, whereas it was at the medium concentration for Na, Fe, Mn, and the high concentration for Ca that collinear DP-LIBS was important. This might be related to the content of the elements themselves in soil, and further research remains to be done. Additionally, the SNR values of SP signals for the low- and medium-concentration samples were close to each other, while there was a significant difference in collinear DP signals. This situation occurred for the element K, as well as Fe, Mn, Na. As for the element Ca, it was the medium and high concentration samples that were able to be distinguished by the SNR of collinear DP signals. Moreover, this result was consistent with the results of spectral analysis. For the RSD of both SP and collinear DP signals, there was no absolute change regulation with concentrations. The value of RSD was lower at high concentrations for all elements, except Ca, which obtained a lower RSD at medium concentrations. These results confirmed that DP signals could be more stable and more suitable for quantitative analysis of elements. Furthermore, the stability of the LIBS signal depends on the element itself and its concentration, and the specific relationship remains to be further studied.

### 3.3. Sensitivity Analysis

#### 3.3.1. Univariate Analysis Models of SP and Collinear DP Signals

In order to intuitively compare the SP and collinear DP signals through elements, all spectral lines of the elements in Table 2 were used to build univariate models. Before modeling, the sample set partitioning based on the joint x-y distance (SPXY) method was used to divide sample sets. Soil samples were split into a calibration set (42 for SP and collinear DP experiments, respectively) and a predication set (21 for SP and collinear DP experiments, respectively) with a ratio of 2:1. For most spectral lines, the correlation between the intensity of both SP and collinear DP signals and the concentration of elements remains to be improved. The spectral lines (K I 518.36 nm, Ca I 445.48 nm, Mg I 517.26 nm Fe I 404.58 nm, Mn I 403.07 nm, Na I 819.47 nm) that obtained the best modeling results were applied for subsequent analysis. In addition, these spectral lines were also used for spectral analysis and stability analysis above. 

**Figure 4:**
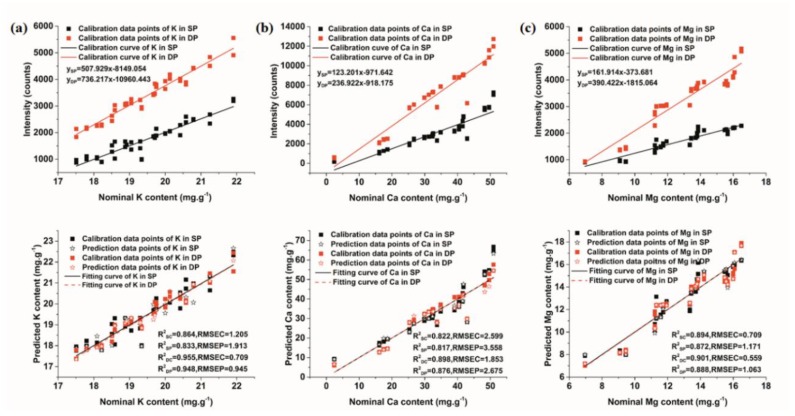

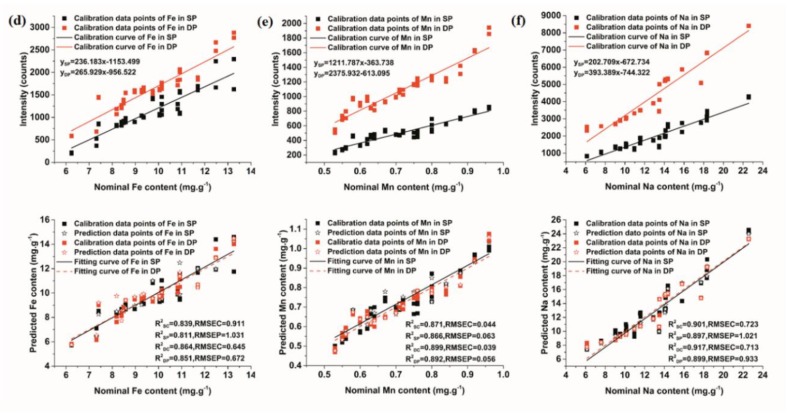
Univariate calibration curves and models of element’s SP and collinear DP signals. (**a**) K; (**b**) Ca; (**c**) Mg; (**d**) Fe; (**e**) Mn; (**f**) Na.

Figure 4 presents the results of univariate models based on these spectral lines. Calibration curves of elements in Figure 4 were firstly fitted using the calibration set, and the predicted content of elements could be obtained by taking the intensity of LIBS signals into the fitting curve. As seen in Figure 4, the slopes of calibration curves of the collinear DP signals were larger than those of the SP signals, which is in alignment with the conclusions of the spectral analysis. The difference in the concentration of elements was relatively obvious when comparing the intensity of collinear DP signals. In addition, univariate models of collinear DP signals showed better performance than those of SP signals, with a higher R^2^_C_ and R^2^_P_ and a lower RMSEC and RMSEP. The preliminary results indicated that collinear DP signals were superior to SP signals for the quantitative analysis of elements. 

LOD can reflect the analytical capabilities of systems and models for elements. According to Equation (1), mentioned above, we calculated the value of LOD for each element based on the calibration curve, and the results are given in Table 3. We can see that the LODs of different elements varied greatly. For all elements, the LODs of collinear DP signals were obviously lower than those of SP signals. For both SP and collinear DP signals, K had the lowest detection limit, while Ca had the highest detection limit. According to the standard of grade of the soil nutrient elements, in which higher grades indicate a shortage of the element, we found that DP-LIBS combined with univariate models could detect the level 5 nutrition standard of k (30–50 ppm), the level 5 nutrition standard of Ca (<300 ppm), and the level 4 nutrition standard of Mg (50–100 ppm). However, the LOD of the LIBS signals is higher than the level 1 nutrition standard of Fe, Mn, Na. The results revealed that collinear DP-LIBS is able to quantify the three macro-nutrients in the range needed for agricultural and environmental purposes, and can provide a reference for the quantitative fertilization of these elements. 

#### 3.3.2. Multivariate Analysis Models of SP and Collinear DP Signals

Because of the complex soil matrix, as well as the rich and overlapped spectral lines of the elements, an analysis focusing only on a single band of elements might result in the loss of valid information, which wouldn’t meet the requirements of the quantitative analysis of elements. In contrast, the multivariate calibration method could make full use of the spectral information, reduce the matrix effect more effectively, and improve the accuracy and repeatability of quantitative analysis. Thus, multivariate analysis methods, including PLSR and LS-SVM, were adopted to evaluate the sensitivity of SP-LIBS and collinear DP-LIBS in the detection of soil elements. In order to avoid overfitting of the model and improve the processing speed at the same time, we selected nearly 200 bands of each element for modeling analysis, which contained the common spectral lines listed in Table 2. Sample sets were the same as those of univariate models.

**Figure 5:**
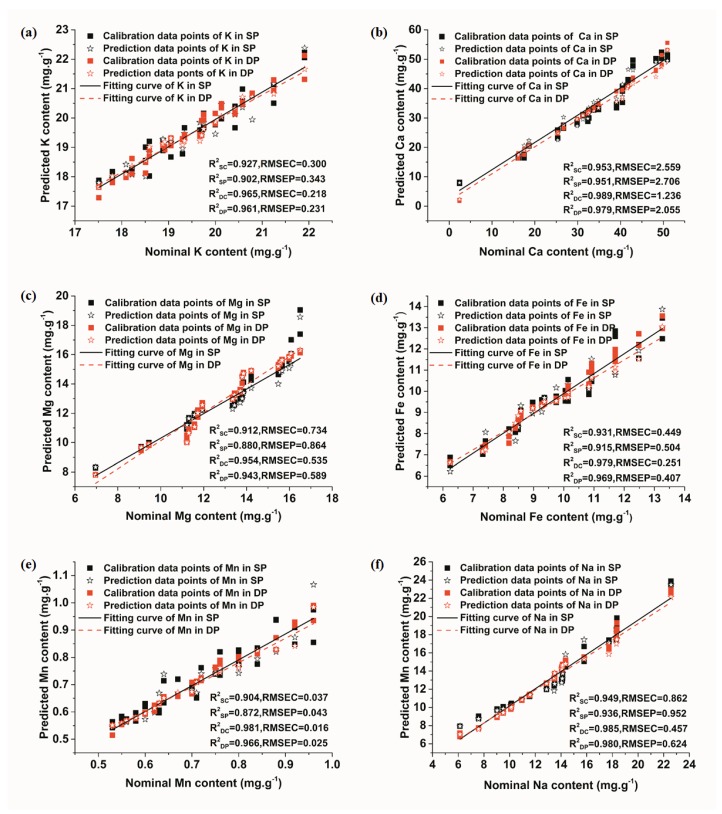
PLSR models of element’s SP and collinear DP signals. (**a**) K; (**b**) Ca; (**c**) Mg; (**d**) Fe; (**e**) Mn; (**f**) Na.

Figure 5a–f shows the calibration and prediction results of PLSR model for K, Ca, Mg, Fe, Mn, and Na, respectively. As seen in Figure 5, for both SP and collinear DP data, most of the calibration and prediction data points were distributed around the fitting curve, indicating that the PLSR model of all elements performed well in predicting their content. In addition, under the condition of collinear DP, R^2^ of calibration sets and prediction sets for all elements were obviously improved, and the RMSEC and RMSCP were reduced. All the values of R^2^ were greater than 0.95, except that the prediction set of the Mg element was 0.9428, which was still acceptable. Additionally, the value of R^2^ of different elements increased in different degrees. The promotion effect for the Mn element was especially obvious, with R^2^ in the prediction set changing from 0.8718 to 0.9664. This might be credited to the concentration of the element. Compared with other elements, the concentration of Mn was relatively low. These results indicate that the detection sensitivity and the prediction accuracy of collinear DP-LIBS were better than that of SP-LIBS. 

LODs for each element based on PLSR models were also calculated, and the results are given in Table 4. It could be seen that LODs of collinear DP signals were obviously lower than those of SP signals. For both SP and collinear DP signals, K had the lowest detection limit, while Ca had the highest detection limit. The results were the same as that of univariate models. According to standard for grade of soil nutrient elements, DP-LIBS combined with PLSR models could satisfy the agricultural and environmental demand for the three macro-nutrients K, Ca and Mg, with LODs below the low nutritional levels of these elements. However, it was still not feasible to quantify the three micro-nutrients in the range needed for agricultural and environmental purposes. 

**Figure 6:**
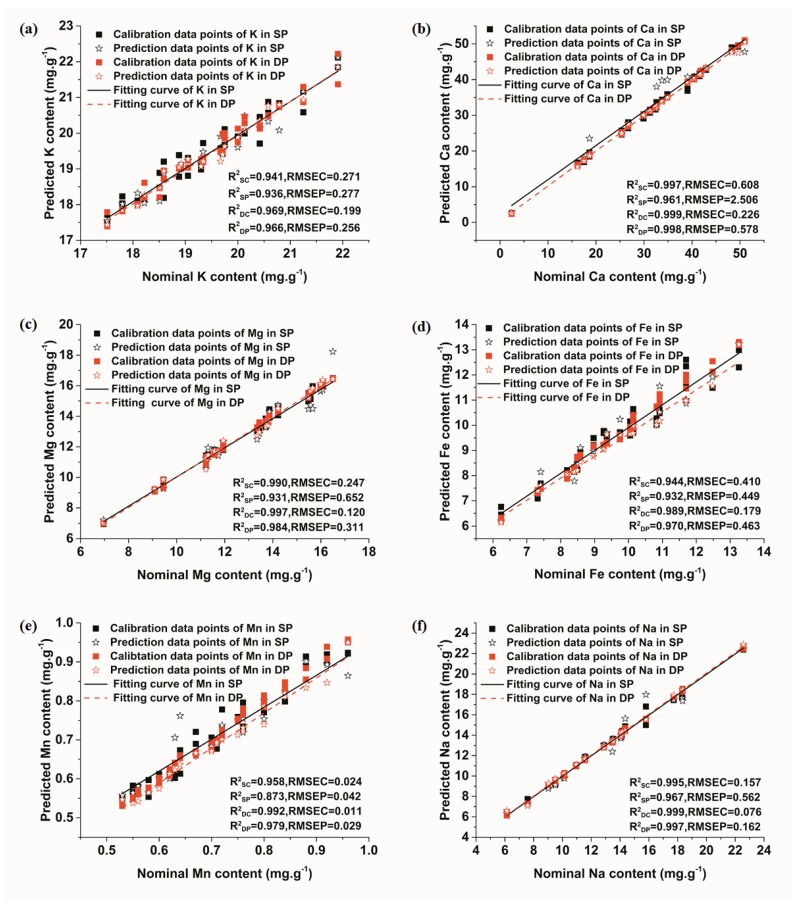
LS-SVM models of elements’ SP and collinear DP signals. (**a**) K; (**b**) Ca; (**c**) Mg; (**d**) Fe; (**e**) Mn; (**f**) Na.

The calibration and prediction results of LS-SVM models for all analyzed elements are provided in Figure 6. It could be observed that the calibration and prediction data points of both SP and collinear DP signals fitted well, indicating that the LS-SVM model had a reliable prediction power for quantitative analysis of soil elements. Moreover, compared with SP-LIBS, the detection sensitivity of collinear DP-LIBS for each element was higher, with all R^2^ being greater than 0.96, higher than those of the SP signals. Meanwhile, for all elements, the value of RMSEC and RMSEP of collinear DP signals were lower than that of SP signals. It was also noticed that the influence degree of collinear DP on the value of R^2^ of each element was different, with the most demonstrable effect being shown on Mn, with R^2^ in the prediction set increasing from 0.8723 to 0.9786. This result was roughly in accordance with that of PLSR model, which further proved that it was the concentration of Mn that mattered. Combining the result of PLSR models, we could conclude that collinear DP-LIBS was more suitable for quantitative analysis of soil elements. 

#### 3.3.3. Comparison of Univariate and Multivariate Analysis Models

In order to investigate the reliability of SP and collinear DP detection sensitivity, we compared the evaluation parameters of three models. The results are stated in Table 5. It could be seen clearly that the value of R^2^ of LS-SVM models for both SP and collinear DP signals were higher than those of PLSR models and univariate models, and the values of RMSEC and RMSEP were the opposite way around. However, the performance of PLSR models was also satisfactory. Moreover, the LODs of elements of PLSR models were lower than that of univariate models, and there was no exact way to calculate the LOD in the LS-SVM model. Taken together, it was the PLSR model that was more suitable for quantitative analysis of elements. In summary, the totality of results of the chemometrics models illustrated that collinear DP-LIBS showed better reliability and sensitivity than SP-LIBS. With the advantages of fast analysis speed, high detection precision, little sample preparation, and multi-element analysis availability, collinear DP-LIBS technique has the potential to be packaged into a field-deployable instrument for real-time and in situ analysis of soil nutrient elements, and provides guidance for precision fertilization in agriculture. High detection limit has always been a difficult problem in the application of LIBS. Since DP-LIBS coupled with the two kinds of models could only deal with the analysis of three macro-nutrients of K, Ca and Mg for practical application, great importance should be attached to the methods of reducing the LODs of elements in future research. Signal enhancement method such as nanoscale signal enhancement, spatial constraint signal enhancement and technology integration can be further studied to improve the ability of quantitative detection of LIBS.

## 4. Conclusions

This research focused on the comparison of the ability of SP-LIBS and collinear DP-LIBS for the quantitative determination of soil nutrient elements. Several aspects, including spectral intensity, signal stability, quantitative analysis models of elements, and the LOD of elements were investigated. It could be found that collinear DP signals had a higher spectral intensity and better signal stability than SP signals. In the aspect of quantitative analysis of elements, chemometrics models of collinear DP signals showed better accuracy and reliability than those of SP signals. Additionally, the LODs of collinear DP signals in univariate models and PLSR models for all elements were lower than those of SP signals. After considering the evaluation indicators of models comprehensively, the PLSR model was regarded most suitable for quantitative analysis of elements, with a satisfactory modeling performance and a relatively low LOD. Generally, collinear DP-LIBS technique combined with chemometrics methods could be a great tool for quantitative analysis of soil nutrient elements. It could therefore provide a theoretical guidance for on-line detection of soil nutrient elements and development of portable instrument. Furthermore, methods of reducing the LOD of LIBS will be the focus of the future work.

## Figures and Tables

**Table 1:** The concentrations (mg·g^−1^) of major nutrient elements in soil samples.

Number	K	Ca	Mg	Fe	Mn	Na
GBW07447	17.51 ± 0.17	48.28 ± 0.71	15.48 ± 0.42	8.58 ± 0.35	0.53 ± 0.01	22.57 ± 0.67
GBW07452	21.91 ± 0.25	29.89 ± 0.57	15.66 ± 0.36	11.70 ± 0.56	0.88 ± 0.02	14.13 ± 0.30
GBW07453	20.58 ± 0.33	2.41 ± 0.14	6.96 ± 0.24	6.24 ± 0.56	0.71 ± 0.01	6.14 ± 0.22
GBW07454	18.92 ± 0.17	50.98 ± 0.71	11.94 ± 0.30	10.14 ± 0.49	0.63 ± 0.02	12.88 ± 0.22
GBW07455	18.09 ± 0.33	32.59 ± 0.50	11.22 ± 0.36	8.40 ± 0.56	0.56 ± 0.02	14.06 ± 0.22
GBW07456	19.67 ± 0.33	34.86 ± 0.50	16.50 ± 0.48	13.26 ± 0.63	0.96 ± 0.04	9.03 ± 0.22

The values are expressed as mean ± SD.

**Table 2:** Spectral emission lines of nutrient elements in soil.

Elements	Emission Lines (nm)	Reference
K	I 404.72, I 518.36, I 766.49, I 769.90	[41,42,43]
Ca	I 445.48, I 616.21, I 643.91	[44]
Mg	I 383.23, I 383.81, I 516.73, I 517.26, I 518.36	[44,45]
Fe	I 404.58, I 406.36, I 428.2, I 428.8	[42,45,46]
Mn	I 279.81, I 403.07, I 403.31, I 403.45	[44,45]
Na	I 818.3, I 819.47	[44,47]

**Table 3:** Comparison of the LODs of elements’ SP and collinear DP signals based on univariate models.

Signal	Parameter	K	Ca	Mg	Fe	Mn	Na
Single pulse	*σ_background_*	8.127	9.487	5.883	10.629	20.196	7.229
	*b*	507.929	123.201	161.914	236.183	1211.787	202.709
	LOD (ppm)	48	231	109	135	50	107
Double pulse	*σ_background_*	7.608	13.820	6.637	11.169	31.679	7.381
	*b*	736.217	236.922	390.422	265.929	2375.932	393.389
	LOD (ppm)	31	175	51	126	40	54

**Table 4:** Comparison of the LODs of elements’ SP and collinear DP signals based on PLSR models.

Signal	Parameter	K	Ca	Mg	Fe	Mn	Na
Single pulse	*a*	25.746	10.405	12.491	8.962	24.680	14.329
	LOD (ppm)	39	96	80	112	41	70
Double pulse	*a*	32.823	12.524	13.398	14.342	26.672	20.130
	LOD (ppm)	30	80	75	70	37	50

**Table 5:** Comparison of three chemometrics models of elements’ SP and collinear DP signals.

Data	Model	Parameter	R^2^_C_	RMSEC	R^2^_P_	RMSEP	LOD (ppm)
Single-pulse of K	Univariate	-	0.864	1.205	0.833	1.913	48
	PLS-DA	7	0.927	0.300	0.902	0.343	39
	LS-SVM	(10,10)	0.941	0.271	0.936	0.277	-
Double-pulse of K	Univariate	-	0.955	0.709	0.948	0.945	31
	PLS-DA	5	0.965	0.218	0.961	0.231	30
	LS-SVM	(10,10)	0.969	0.199	0.966	0.256	-
Single-pulse of Ca	Univariate	-	0.822	2.599	0.817	3.558	231
	PLS-DA	10	0.953	2.559	0.951	2.706	96
	LS-SVM	(8,10)	0.997	0.608	0.961	2.506	-
Double-pulse of Ca	Univariate	-	0.898	1.853	0.876	2.675	175
	PLS-DA	8	0.989	1.236	0.979	2.055	80
	LS-SVM	(9,9)	0.999	0.226	0.998	0.578	-
Single-pulse of Mg	Univariate	-	0.894	0.709	0.872	1.171	109
	PLS-DA	5	0.912	0.734	0.880	0.864	80
	LS-SVM	(3,7)	0.990	0.247	0.931	0.652	-
Double-pulse of Mg	Univariate	-	0.901	0.559	0.888	1.063	51
	PLS-DA	6	0.954	0.535	0.943	0.589	75
	LS-SVM	(10,10)	0.997	0.120	0.984	0.311	-
Single-pulse of Fe	Univariate	-	0.839	0.911	0.811	1.031	135
	PLS-DA	9	0.931	0.449	0.915	0.504	112
	LS-SVM	(5,4)	0.944	0.410	0.932	0.449	-
Double-pulse of Fe	Univariate	-	0.864	0.645	0.851	0.672	126
	PLS-DA	8	0.979	0.251	0.969	0.407	70
	LS-SVM	(7,9)	0.989	0.179	0.970	0.463	-
Single-pulse of Mn	Univariate	-	0.871	0.044	0.866	0.063	50
	PLS-DA	7	0.904	0.037	0.872	0.043	41
	LS-SVM	(10,10)	0.958	0.024	0.873	0.042	-
Double-pulse of Mn	Univariate	-	0.899	0.039	0.892	0.056	40
	PLS-DA	11	0.981	0.016	0.966	0.025	37
	LS-SVM	(10,10)	0.992	0.011	0.979	0.029	-
Single-pulse of Na	Univariate	-	0.901	0.723	0.897	1.021	107
	PLS-DA	9	0.949	0.862	0.936	0.952	70
	LS-SVM	(7,7)	0.995	0.157	0.967	0.562	-
Double-pulse of Na	Univariate	-	0.917	0.713	0.899	0.933	54
	PLS-DA	7	0.985	0.457	0.980	0.624	50
	LS-SVM	(8,10)	0.999	0.076	0.997	0.162	-
